# Atypical Presentation of Renal Leiomyosarcoma: A Case Report

**DOI:** 10.7759/cureus.5433

**Published:** 2019-08-19

**Authors:** Danny Darlington, Fatima Shirly Anitha

**Affiliations:** 1 Urology, Pondicherry Institute of Medical Sciences, Pondicherry, IND; 2 Pediatrics, Church of South India Kalyani Multispeciality Hospital, Chennai, IND

**Keywords:** malignant hypertension, primary renal leiomyosarcoma, renal sarcoma

## Abstract

Primary renal sarcomas are rare comprising from 0.8 to 2.7% of adult renal tumours. They cannot be distinguished clinically or radiologically from the more common renal cell carcinomas. Leiomyosarcoma is the most common histological subtype accounting for around 50-60% of renal sarcomas. Leiomyosarcomas arise from the renal capsule, renal vein or the renal pelvis. They present with nonspecific clinical symptoms and signs and usually have a dismal outcome. Rarely atypical presentations such as acute tumour rupture and hematuria are encountered. We report a 27-year-old woman who presented with malignant hypertension. The hypertension was controlled emergently with antihypertensive agents. In view of the young age, a thorough workup was conducted to identify the cause of hypertension. Imaging studies revealed a solid enhancing renal tumour compressing the renal vasculature. As the staging workup did not reveal any evidence of metastases, the patient underwent right-sided open radical nephrectomy after adequate control of hypertension. The histological examination uncovered the renal tumour to be a leiomyosarcoma. Postoperative period was uneventful and she was doing well on one year follow up. Surprisingly the blood pressure normalised postoperatively and the patient was weaned off of her antihypertensive medications. This case is presented to highlight the atypical acute presentation of primary renal leiomyosarcoma with relatively good prognosis. Timely diagnosis and meticulous surgical resection improved the prognosis of this aggressive renal malignancy.

## Introduction

Primary renal sarcomas are rare malignant tumours of the kidney with poor prognosis. Leiomyosarcomas account for the majority of renal sarcomas comprising 50-60% of these aggressive tumours [1]. They occur in the elderly and are usually associated with poor prognosis because of the rapid growth and delayed presentation. Renal capsule, renal vein, and renal pelvis are the common sites of origin of renal leiomyosarcomas. The clinical signs and symptoms are indistinguishable from renal cell carcinoma. Rarely these tumours can present in an atypical fashion. Acute presentations in leiomyosarcomas are rare and limited to spontaneous tumour rupture and hematuria [2]. We report a case of primary renal leiomyosarcoma in a 27-year-old woman who presented with malignant hypertension. The tumour could not be distinguished from renal cell carcinoma either clinically or radiologically. However, a thorough histological examination revealed the final diagnosis. Our case report is the first instance of a renal leiomyosarcoma presenting with malignant hypertension.

## Case presentation

**Figure 1:**
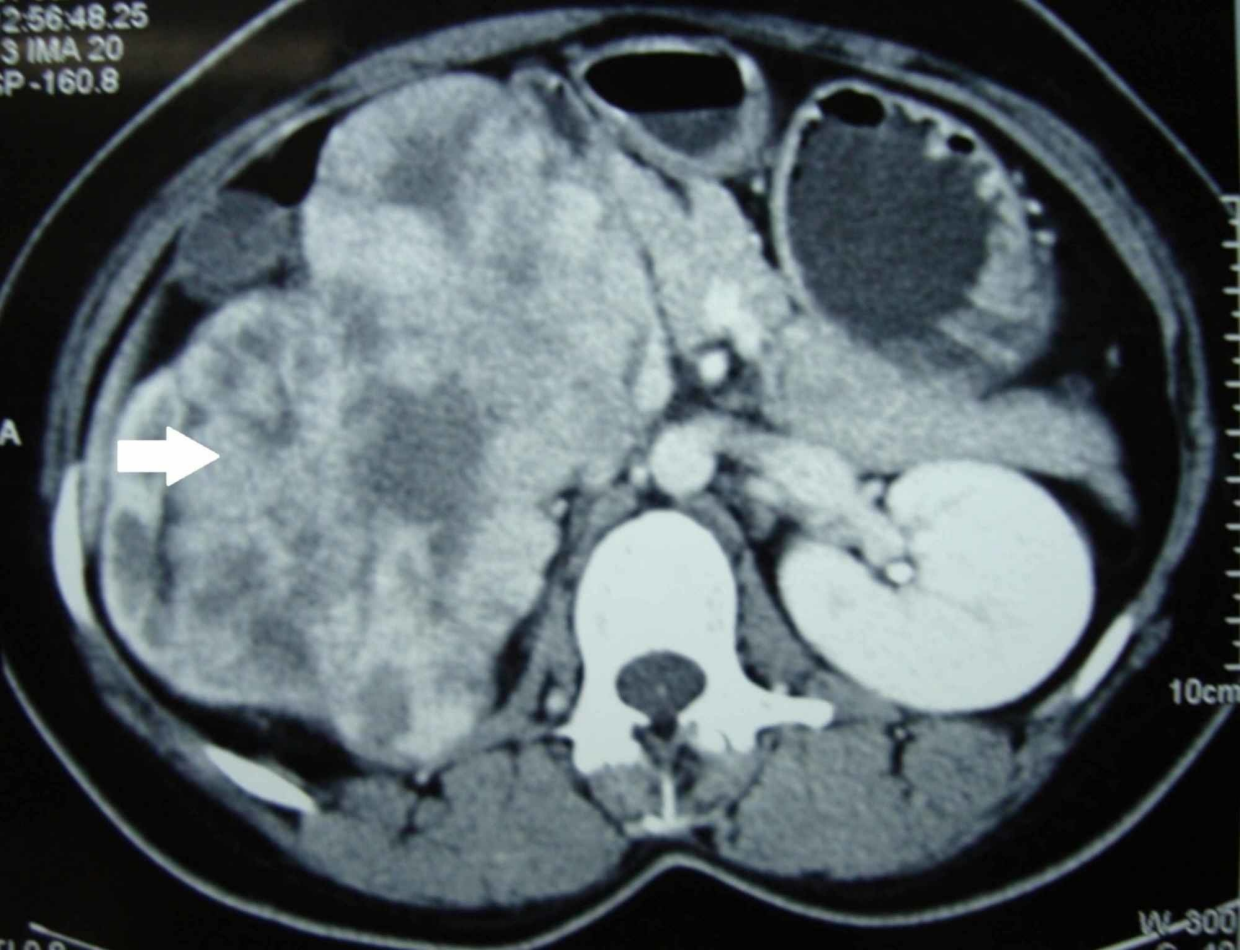
Contrast-enhanced computed tomogram showing the enhancing right renal mass (arrow)

**Figure 2:**
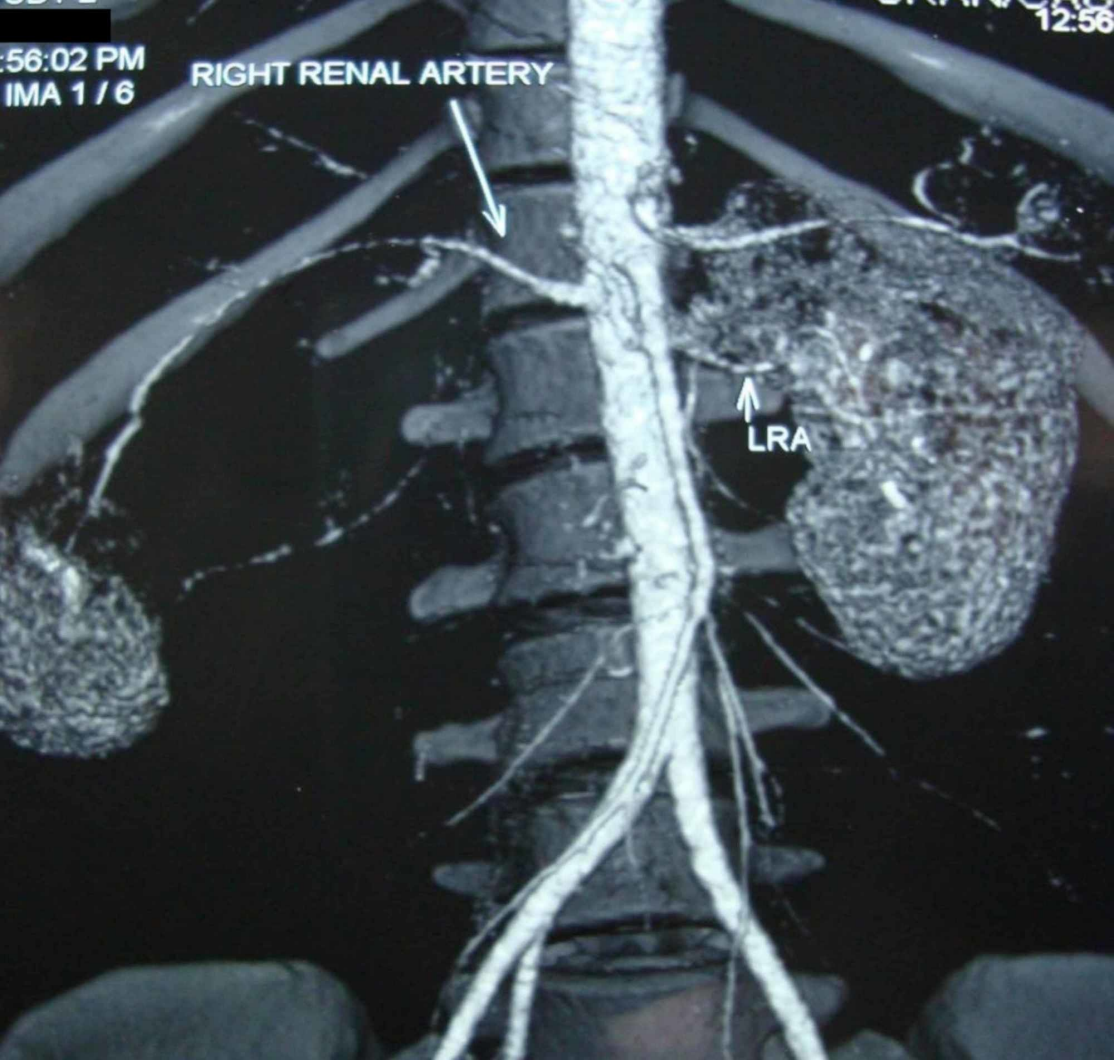
Reconstructed image of the contrast-enhanced computed tomogram depicting the tumour compressing right renal artery (long arrow)

A 27-year-old previously normotensive woman presented to the medical emergency department with sudden onset persistent headache. She neither had similar symptoms in the past nor was she a drug abuser. She was unmarried and had no other illness. On examination, she was conscious and oriented. She was not anaemic or jaundiced. She had no significant past medical history or abdominal surgery and her family history was insignificant. Her blood pressure was 200/160 mm of mercury at presentation and examination of her fundus revealed grade one hypertensive retinopathy. Abdominal examination revealed a mobile mass involving the right lumbar and right hypochondriac regions reaching the midline. The firm mass was moving with respiration, nontender and bimanually palpable. There was no other palpable abdominal mass. Examination of the chest and spine was normal. Her blood investigations including renal function test, blood counts, liver function tests, serum calcium, sodium, potassium, and erythrocyte sedimentation rate were within normal limits. Plasma free metanephrine levels also were normal. Urine examination revealed microalbuminuria and the presence of eight red blood cells per high power field. Ultrasonographic examination of the abdomen was done which revealed a 15x10x7 cm sized solid renal mass with internal vascularity occupying the upper pole and interpolar regions. Echocardiography showed normal cardiac chambers. Computed tomography (CT) of the brain was normal. The metastatic workup was completed with contrast-enhanced computed tomography (CECT) of the abdomen and a chest roentgenogram. CECT of the abdomen revealed an enhancing tumour 15x10x7 cm in size arising medially from the right kidney and displacing it laterally and inferiorly (Figure 1). The tumour was closely related to the inferior vena cava (IVC) and compressing the renal vessels (Figure 2). The inferior vena cava was devoid of any thrombus. There was no evidence of paraaortic lymphadenopathy, ascites or liver metastases. The adrenal glands were normal and chest roentgenogram did not show any evidence of metastases.

**Figure 3:**
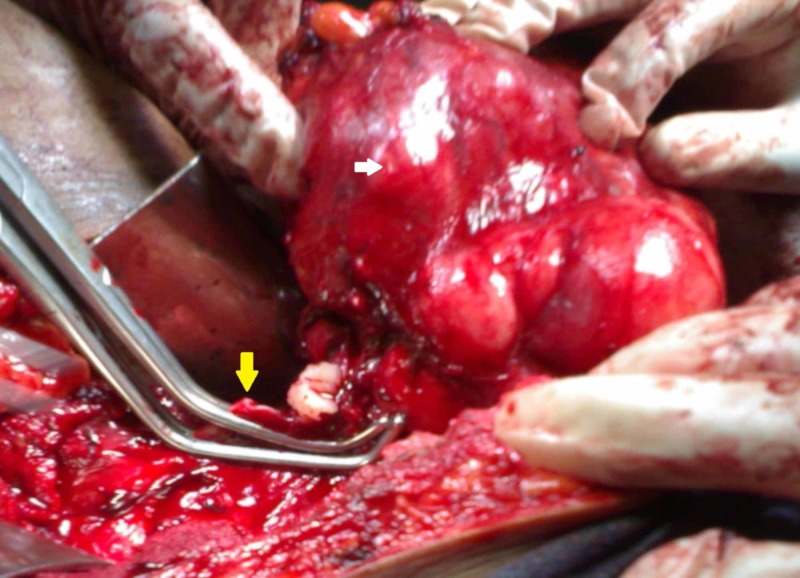
Intraoperative image showing the tumour (white arrow) closely related to the inferior vena cava (yellow arrow) and removed with a cuff of it

**Figure 4:**
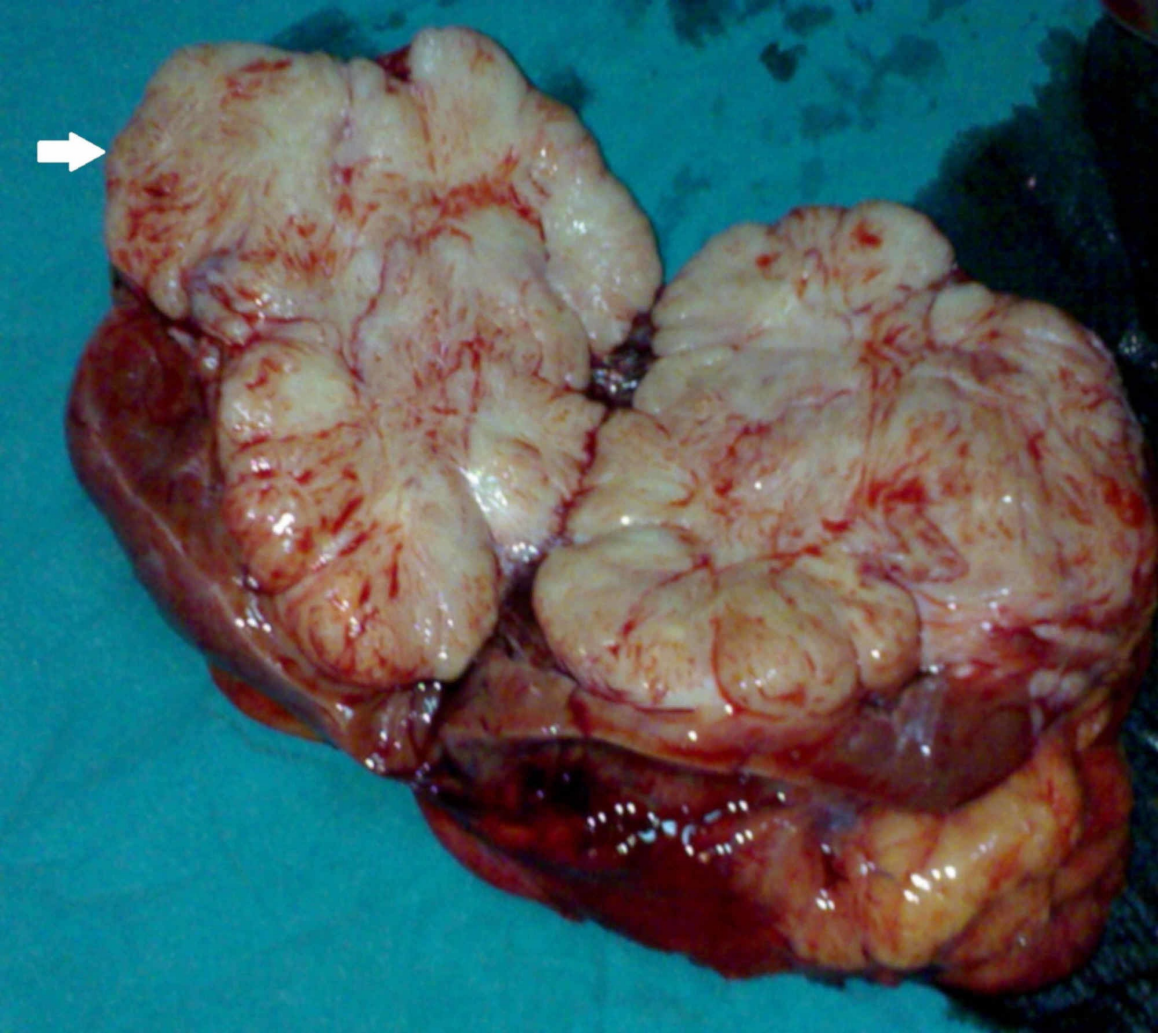
Cut section of the radical nephrectomy specimen with tumour occupying the upper and interpolar regions of the kidney and exhibiting whorled appearance (arrow)

**Figure 5:**
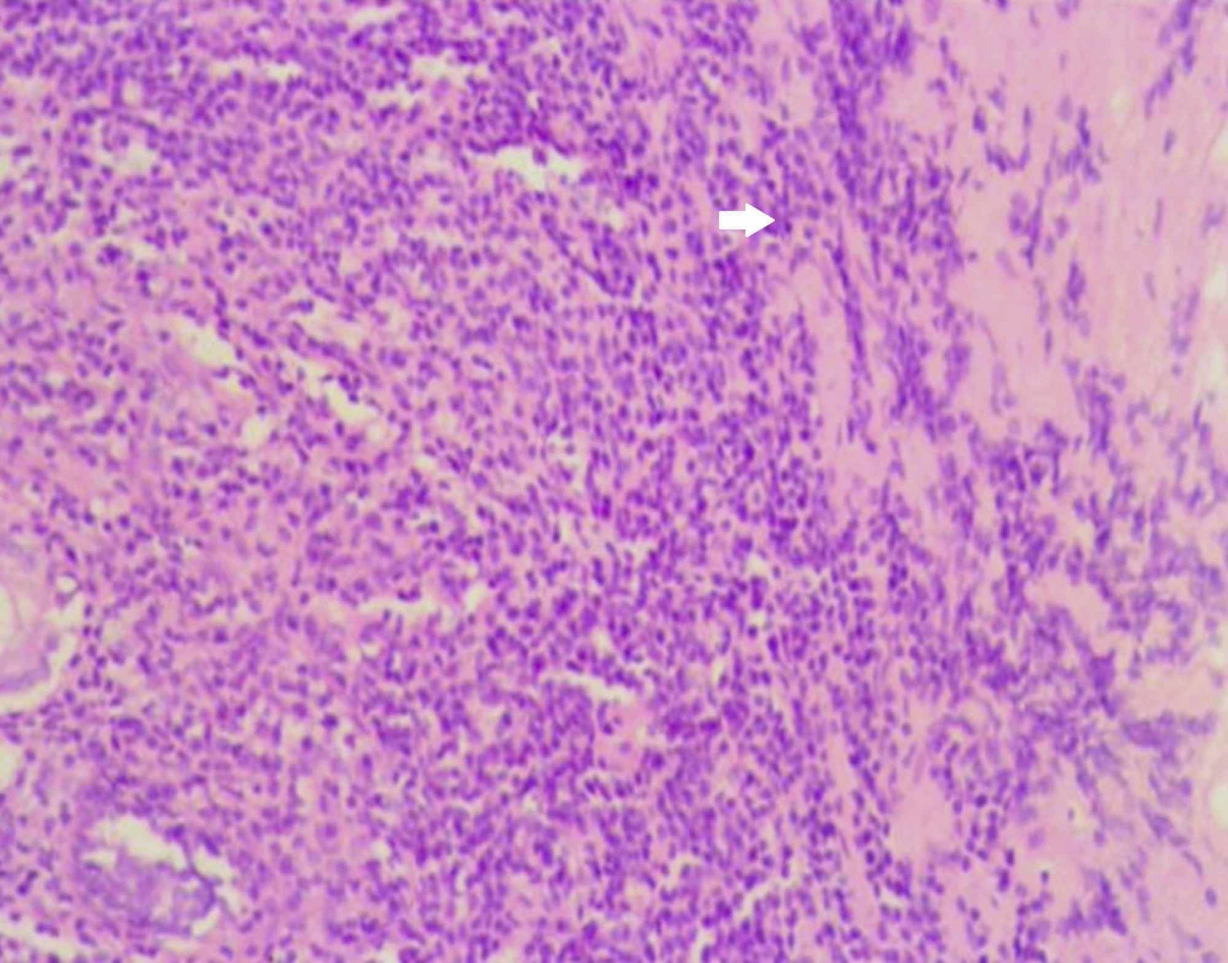
Histopathological image showing the tumour composed of spindle cells with pleomorphic nuclei as indicated by the arrow (hematoxylin and eosin stain, 40x magnification)

**Figure 6:**
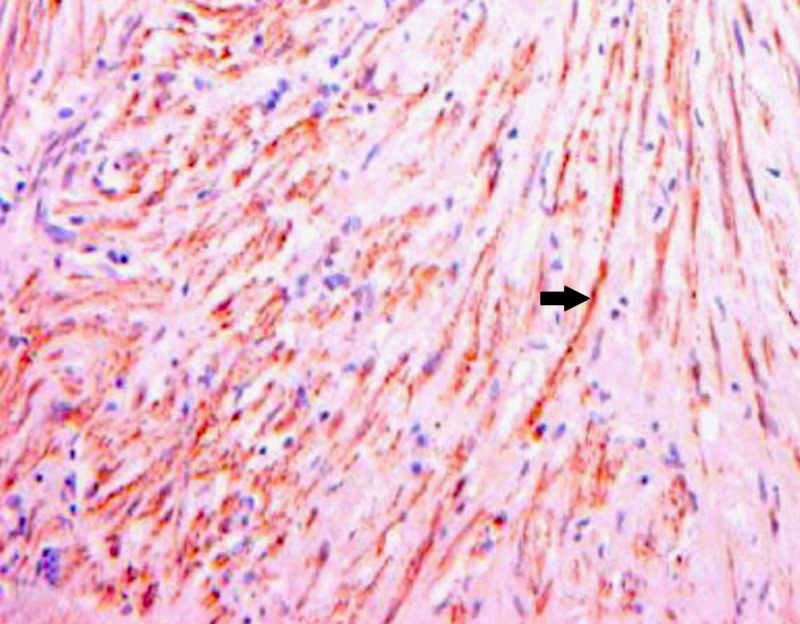
Immunohistochemical staining for smooth muscle actin demonstrating uniform positive staining by tumour cells as indicated by the arrow (40x magnification)

The hypertension was controlled with intravenous nitroprusside infusion followed by double oral antihypertensive agents (nifedipine 30 mg once daily and atenolol 50 mg twice daily). The patient underwent right-sided radical nephrectomy through a flank incision and the tumour was approached extrapleurally and extraperitoneally after excising the eleventh rib. It was involving the upper and interpolar regions and closely abutting the renal vessels and the IVC. There was no regional lymphadenopathy. There was no episode of blood pressure fluctuation during or after the procedure. In view of the tumour being adherent to the renal vein and its ostium, nephrectomy was performed along with the removal of the cuff of IVC (Figure 3). Cut section of the specimen showed a fleshy tumour with whorled appearance replacing the upper and interpolar regions of the right kidney (Figure 4). Histopathological examination showed the tumour to be composed of spindle cells with elongated cigar-shaped nuclei showing moderate pleomorphism and ten mitotic figures per ten high power fields (Figure 5). Necrosis was seen to involve around 25% of the tumour volume. Lymphovascular invasion was absent and perinephric fat was free of the tumour. Immunohistochemical study showed strong positivity for smooth muscle actin (SMA) and desmin and negativity for cytokeratin and S100 (Figure 6). Postoperative period was uneventful and her blood pressure then was controlled with a single antihypertensive agent (nifedipine 30 mg once daily). The pathological staging was pT3N0M0 and hence the patient was not given any adjuvant chemotherapy. She was on regular follow up and a month later was weaned off all her antihypertensive medications. The patient is recurrence-free and is maintaining normal blood pressure for the last one year on follow up.

## Discussion

Primary leiomyosarcoma of the kidney is an extremely rare entity. It usually occurs in elderly women and carries a poor prognosis [3]. Renal leiomyosarcomas show rapid growth characteristics and are usually detected late in the disease process. These aggressive tumours possibly arise from the renal pelvis, renal vein or the renal capsule and rarely from the inferior vena cava [4-5]. In general leiomyosarcomas are solitary lesions which occur in the fourth to the sixth decade of life. The present case is unique in that it occurred in a 27-year-old female and is the second youngest reported so far in the literature. However, there was no family history of malignancy or genetic conditions predisposing to malignancies. Sevilla et al. report a case of renal leiomyosarcoma in a 16-year-old adolescent with tuberous sclerosis [6].

Renal leiomyosarcomas usually present with nonspecific symptoms like weight loss, anaemia, bone pain, and hematuria and symptoms usually indicate locally advanced disease [7]. They can present as a solid or cystic renal lesion and cannot be clinically or radiologically differentiated from renal cell carcinoma [8]. In fact, the cystic variant of leiomyosarcoma is associated with grave prognosis [9]. The common sites of metastases include lungs and liver however rare instances of metastases to the opposite kidney and soft tissues have been reported [10-11].

Moazzam et al. report a case of renal leiomyosarcoma presenting with spontaneous retroperitoneal haemorrhage and hypotension [2]. However renal leiomyosarcoma presenting with malignant hypertension has never been reported in the literature. The probable pathophysiological mechanism of hypertension in our case could be the tumour compressing the renal artery and activating the renin-angiotensin axis.

Imaging features of leiomyosarcoma are similar to renal cell carcinoma. It presents as an enhancing renal mass with or without necrosis. A cystic component may be present in large tumours. Renal cysts with thick walls must raise the suspicion of sarcoma [9]. Histological examination is the mainstay of diagnosis of leiomyosarcomas. Leiomyosarcomas exhibit fascicles of spindle-shaped cells with pleomorphic nuclei and mitotic figures under the microscope. Necrosis is often present and is considered an important prognostic factor. The paramount aim is to differentiate leiomyosarcoma from leiomyoma, renal cell carcinoma with sarcomatoid differentiation and epithelioid angiomyolipoma. Leiomyosarcomas can be easily differentiated from leiomyomas. Though nuclear pleomorphism can be seen in both, necrosis is present only in the malignant counterpart [8].

While leiomyosarcomas contain predominantly monomorphic cells, sarcomatoid renal cell carcinomas contain pleomorphic cells and lack the typical fascicles of smooth muscle cells seen in leiomyosarcoma. If the epithelial component is present, the diagnosis is in favour of a sarcomatoid renal cell carcinoma than leiomyosarcoma. Immunohistochemical positivity for cytokeratin confirms the diagnosis of a sarcomatoid variant of renal cell carcinoma [8]. Epithelioid angiomyolipoma is another renal neoplasm which mimics leiomyosarcoma histologically however it can be distinguished from leiomyosarcoma by positivity for melanocytic markers like S100 in immunohistochemistry [12]. In general, leiomyosarcoma shows strong positivity for smooth muscle actin and desmin and negativity for cytokeratin and S100.

Radical surgery is the definitive treatment modality for renal leiomyosarcomas. In our case, we resorted to open radical nephrectomy as the tumour was huge almost replacing the kidney. Meticulous and complete surgical resection is the single best treatment for this aggressive renal tumour. The five-year survival approaches 60% when surgical margins are negative [3]. Adjuvant chemotherapy and radiotherapy have no role in the completely resected tumours although they can be tried after incomplete resections. There is no standardised protocol of chemotherapy or radiotherapy for this rare renal malignancy. Drugs such as gemcitabine, cyclophosphamide, vincristine, adriamycin, and dacarbazine have been used however the survival benefit is inconclusive [1]. Recent studies have shed light on the effectiveness of tyrosine kinase inhibitors like sunitinib in treating leiomyosarcomas [13].

The five-year survival for renal leiomyosarcomas is 39% which is relatively lower compared to leiomyosarcomas in other locations. Complete resectability is the main prognostic factor followed by histological factors including tumour grade, nuclear grade and necrosis occupying more than 50% of the tumour. Tumour size more than 5 cm is a poor prognostic factor as is the presence of systemic metastases [1]. Our patient had necrosis occupying less than 50% of the tumour, low nuclear grade and complete tumour resection which explains the better prognosis.

## Conclusions

This case has been reported to highlight the rare presentation of renal leiomyosarcoma underscoring the significance of thoroughly investigating patients with young age hypertension. Though regarded to be aggressive renal tumours, surgical resectability is an important modifiable prognostic factor which can improve the outcome. Newer modalities of treatment are on the way but at present surgery is the only hope for these unfortunate patients.
